# Elucidation and Regulation of Polyphenols in the Smoking Process of Shanxi Aged Vinegar

**DOI:** 10.3390/foods10071518

**Published:** 2021-07-01

**Authors:** Sankuan Xie, Jia Song, Bingqian Fan, Xuan Li, Yingqi Li, Fangming Mou, Yu Zheng, Min Wang

**Affiliations:** Key Laboratory of Industrial Fermentation Microbiology Ministry of Education, Tianjin Engineering Research Center of Microbial Metabolism and Fermentation Process Control, State Key Laboratory of Food Nutrition and Safety, College of Biotechnology, Tianjin University of Science and Technology, Tianjin 300457, China; xskuan@mail.tust.edu.cn (S.X.); tjsongjia@tust.edu.cn (J.S.); fanbingqian96@mail.tust.edu.cn (B.F.); lixuan9853@mail.tust.edu.cn (X.L.); liyingqi97@mail.tust.edu.cn (Y.L.); mfm2019@mail.tust.edu.cn (F.M.); yuzheng@tust.edu.cn (Y.Z.)

**Keywords:** Shanxi aged vinegar, polyphenols, smoking, influencing factors, in situ regulation

## Abstract

Polyphenols (PPs) are the main contributors to the health functions of Shanxi aged vinegar (SAV) and are mainly produced during the smoking process. This study aimed to explore the feasibility of regulating the accumulation of total water-soluble PPs (TWSP) by changing environmental factors based on the distribution of PPs. A total of eleven PPs, such as vanillin, vanillic acid, and (e)-ferulic acid, were detected during the smoking process. During the smoking process, the content of TWSP gradually increased and was accompanied by changes in environmental factors. Spearman correlation analysis and verification experiments showed that temperature, amino acids, and reducing sugars, as the main influencing factors, promoted the accumulation of TWSP. The in situ regulation strategy of changing environmental factors significantly increased the accumulation of TWSP by 12.24%.

## 1. Introduction

Polyphenols (PPs) are a large collection of at least 10,000 different compounds that contain one or more benzene rings and different numbers of hydroxyl, carbonyl, and carboxyl groups [1,2]. PPs have the functions of antioxidation, lipid metabolism regulation, blood pressure control, cardiovascular disease prevention, liver protection, and antitumor activities, which are the main contributors to the health functions of Shanxi aged vinegar (SAV) [3].

PPs are affected by various environmental factors. PPs are often bonded to matrix molecules, and this binding is mainly noncovalent bonds and sometimes covalent bonds [4,5,6,7]. Ionic strength, temperature, pH, and moisture have important effects on the combination of PPs and the matrixes [8,9] that ultimately affect the content of PPs in the environment. PPs are also sensitive to the environment. For example, tea polyphenols are sensitive to temperature and are easily degraded at high temperatures [10]. Proanthocyanidins are sensitive to acid, whereas anthocyanins are produced by chain breaks under the action of acid catalysis [11]. The accumulation and succession of PPs are also related to the substrate composition in the reaction systems. Some compounds, such as tyrosine, can act as precursors to promote the accumulation of PPs [12]. Other compounds, such as methylglyoxal [13] and glyoxylic acid [14], may provide functional groups in the derivation of PPs to promote the succession of PPs. In addition, hydroxylation, methylation, and methoxylation also have an important impact on the succession of PPs [15,16]. Food processing often causes changes in a variety of environmental factors that affect the accumulation of PPs, which makes the formation mechanism of PPs complicated and difficult to regulate. 

PPs in vinegar are mainly related to raw materials and processes [3], and the distribution of PPs differ in various types and production stages of vinegar. Ren et al. [17] compared the distribution of PPs in SAV and Zhenjiang aromatic vinegar. Seven PPs with different types and contents were detected in SAV and Zhenjiang aromatic vinegar. Chen et al. [18] detected eight PPs during the aging process of SAV, and they found that the content of PPs was remarkably different at different aging times. As many as 41 PPs have been detected in the 8-year-old SAV, among which the main PPs are 3-(4-hydroxy-3-methoxyphenyl) propionic acid, vanillin, and vanillic acid [19]. The main sources of PPs are grains, nuts, seeds, fruits, beverages, vegetables, and spices [2]. Food processing changes various environmental factors, which, in turn, affect the types and content of PPs. For example, cooking makes petunidin, malvidin, and delphinidin, which are sensitive to temperature, almost completely disappear [20]. Owing to changes in temperature, moisture, and drying time, substrate drying also considerably affects the content of many PPs [10]. SAV uses grains, including sorghum, peas, wheat bran, rice hull, and millet bran, as raw materials, and its brewing process generally involves alcohol fermentation, acetic acid fermentation (AAF), smoking, and aging. AAF begins when the mash formed by alcohol fermentation is mixed with wheat bran, rice hull, and millet bran (the moist mixture is called Pei, and it is also called Cupei in the AAF stage). Smoking is carried out in a smoking furnace with a fire channel underneath and a smoking pot on the top. Put the Cupei at the end of AAF (mature Cupei) into the smoking pot to start the smoking (Pei is often called smoking Pei in the process of smoking). Transfer the smoking Pei to the adjacent pot every day, and the turning during the transfer process will make the Pei mixed and heated evenly. Smoking is a high-temperature brewing process dominated by Maillard reaction and the main stage of polyphenol accumulation [21]. Dynamic changes in temperature, acidity, moisture, and other environmental factors in the process of smoking affect the accumulation of PPs. However, knowledge of the distribution of PPs in the process of smoking is limited, and research on the regulation of PPs in complex systems is lacking. 

In this study, the distribution of PPs during the smoking process was revealed via gas chromatography–mass spectrometry (GC–MS). The correlation between PPs and environmental factors was analyzed and verified. On the basis of this information, a strategy for regulating total water soluble polyphenols (TWSP) in smoking was proposed. The potential mechanism by which environmental factors affect the accumulation of PPs was also discussed. 

## 2. Materials and Methods

### 2.1. Sample Collection

Samples of SAV were collected from Taiyuan, China. Both the mature Cupei (the sample at the end of AAF) and the smoking Pei were obtained from about 30 cm from the upper surface of the pot (height and width are 82 and 77 cm, respectively). A five-point sampling method was used for sampling on a 30 cm cross-section. The five-point samples taken from one pot were mixed uniformly to obtain a uniform sample. The smoking Pei samples were collected from the 1st to the 5th day of the smoking process. A sufficient amount of mature Cupei was taken, and each sample of smoking Pei was about 200 g. Each sample was set in triplicate. The samples were transported in an ice box and stored in a refrigerator, at −80 °C.

### 2.2. Determination of TWSP and Physicochemical Indexes 

Prior to testing physicochemical indexes, solid samples were preprocessed to obtain extracts. After adding 45 mL of water to 5 g of the solid sample, the mixture was shaken at room temperature for 3 h and centrifuged at 1700× *g* for 10 min (Centrifuge TG16-WS, Xiangyi Co., ltd., Changsha, China). The supernatant was collected for analysis. The utilizing optimized Folin–Ciocalteu assay was used to determine the TWSP content [21]. The pH was measured with a pH meter S20P (Mettler Toledo Co., ltd., Shanghai, China). Total acid was evaluated by titration using standardized solution (0.1 mol/L sodium hydroxide) with phenolphthalein as indicator. Amino nitrogen was determined by the ninhydrin method of the European Brewing Convention. Changes in pH and contents of total acid and amino nitrogen during the brewing process were detected after following previously described methods [22]. Content of total reducing sugars was determined via Fehling’s test. Amino acids were qualitatively and quantitatively detected by using an amino acid analyzer S433D (Sykam Co., ltd., Eresing, Germany), following the manufacturer’s instructions. The temperature of the sampling point was measured with a thermometer. The moisture content in the smoking Pei was determined via the drying method. The reducing sugars in the samples were determined by high-performance liquid chromatography [23] (Agilent 1260, Agilent Technologies Inc., Santa Clara, CA, USA).

### 2.3. Analysis of PPs Composition 

The composition of PPs in the smoking samples were detected via GC–MS. First, 10 mL of the supernatant obtained in Section 2.2 was collected, and PPs were extracted by following a previously described method [19]. After that, 2, 4, 5-trihydroxybenzoic acid (3B Scientific Co., ltd., Libertyville, IL, USA) was used as an internal standard, and 0.002 g was added to 10 mL of the extracts during pretreatment. Then 1 mL of bis-(trimethylsilyl) trifluoroacetamide (BSTFA; Supelco, Bellefonte, PA, USA) +1% trimethylchlorosilane (Aladdin Bio-Chem Technology Co., ltd., Shanghai, China) was added to the PP extracts, and then it was reacted for 3 h, at 70 °C, in a water bath (thermostat water bath DK-8D, Qiaofeng Co., ltd., Shanghai, China). An HP-5 column (30 m length × 250 μm internal diameter × 0.25 μm film thickness; Agilent, Agilent Technologies Inc., Santa Clara, CA, USA) attached to an Agilent 6890 N series gas chromatograph that was connected to a 5973 mass selective detector (Agilent, Agilent Technologies Inc., Santa Clara, CA, USA) was utilized for the qualitative and quantitative analysis of PPs. The temperature of both the injection port and the detector was 250 °C. The heating program of the oven was set to start at 100 °C and increase to 280 °C at a rate of 3 °C/min. Mass spectra were obtained at 70 eV through the electron ionization method. Compounds were identified by comparing their retention times and mass spectra with those of authentic standards. Their concentrations were calculated by comparing their peak areas with those of internal standard compounds.

### 2.4. Influence of Environmental Factors on TWSP

In the laboratory, the influence of environmental factors on TWSP was analyzed through single factor experiments. Smoking is the process of heat-treating mature Cupei. In the single-factor experiment conducted in the laboratory, the water bath heating method was used to control the heat treatment temperature of the mature Cupei. A 500 mL Erlenmeyer flask containing 300 g of mature Cupei was heated in a water bath. In the temperature single factor experiment, the temperature of the control group (CG) for 1, 2, 3, 4, and 5 days was set to 55, 85, 90, 95, and 90 °C, which was close to the temperature in production. The temperature settings of different experimental groups of 1–5 days were experimental group 1 (EG 1; 65, 85, 90, 95, and 90 ℃), experimental group 2 (EG 2; 75, 85, 90, 95, and 90 ℃), experimental group 3 (EG 3; 85, 85, 90, 95, and 90 °C), and experimental group 4 (EG 4; 95, 95, 95, 95, and 95 °C). In the single-factor experiment with exogenous addition, the control group (CG) was mature Cupei, and the temperature for 1–5 days was set at 55, 85, 90, 95, and 90 °C. In the experimental group, pure amino acids and reducing sugars were mixed together and added into mature Cupei, and the temperature setting was the same as that of the control group. The effects of amino acids and reducing sugars were assessed by setting the gradients of the amounts of amino acids and reducing sugars added to 0.5, 1.0, 1.5, 2.0, and 2.5 × of the initial concentration of the corresponding substances.

### 2.5. Data Analysis Methods

Spearman correlation analysis was performed to analyze the correlation between environmental factors and PPs by using SPSS Statistics (19.0). Correlation heat maps were drawn by using the Multiple Experiment Viewer software (4.9.0).

## 3. Results

### 3.1. Distribution of PPs and Changes of TWSP Content during the Smoking Process

**Figure 1:**
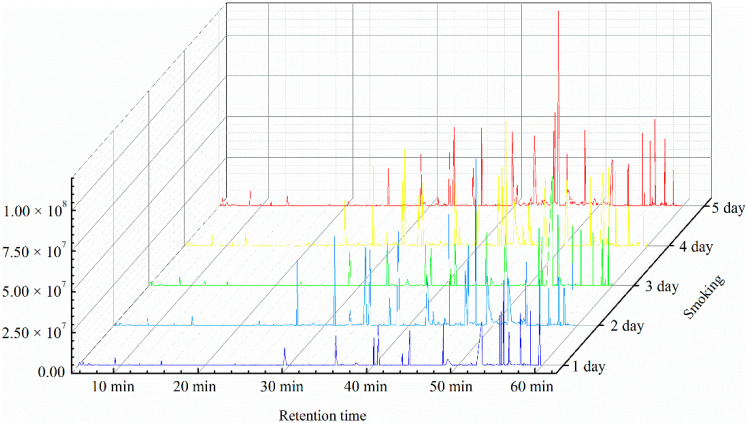
Gas chromatography–mass spectroscopy total ion diagram during the smoking process of Shanxi aged vinegar.

PP composition during the smoking process was analyzed via GC–MS. The diagram of total ion current is shown in Figure 1. From the 1st day to the 5th day of smoking, the number of substances detected was 598 ± 8, 712 ± 23, 745 ± 41, 659 ± 12, and 618 ± 25, respectively. The number of various types of substances detected initially increased and then decreased. The PPs in the detected compounds were analyzed (Table 1). Eleven PPs were detected during the smoking process of SAV: (1) vanillin, (2) vanillic acid, (3) (e)-ferulicacid, (4) (e)-4-hydroxycinnamic acid, (5) 4-hydroxybenzoic acid, (6) (3R,4R)-dihydro-3,4-bis[(3-hydroxy-4-methoxyphenyl) methyl]-2(3H)-furanone, (7) vanillinmandelic acid, (8) 1-(3-hydroxy-4-methoxyphenyl)-1,2-ethanediol, (9) 3,5-dihydroxybenzoic acid, (10) (e)-3-(3-hydroxyphenyl) acrylic acid ethyl ester, and (11) dihydroferulic acid. The types and contents of PPs in the smoking samples at different times were different. The types of PPs gradually increased from six on the 1st day to nine on the 5th day. PPs 1–4 were detected during the entire smoking process. PPs 5, 6, and 9 were observed in the middle and late stages of smoking. PPs 10 and 11 only appeared on the 4th and 3rd day of the smoking process, respectively (Table 1). The content of the PPs dynamically changed during the smoking process. The content of PPs 2, 3, 5, and 9 gradually increased with smoking time, whereas the content of PP 7 initially decreased and then increased. The content of PPs 4, 10, and 11 initially increased and then decreased. The content of PPs 1, 6, and 8 did not exhibit a particular trend, but their content at the end of the smoking process was higher than that at the beginning. The distribution of PPs in the samples at the end of the smoking process (5th day) was analyzed. Among the nine PPs, the proportion of PPs 1, 2, 3, 6, and 9 in TWSP was 6.44%, 11.84%, 33.56%, 22.69%, and 14.40%, respectively. These PPs were the main components in smoking Pei, and their total proportion was 88.92%. Among these PPs, PP 3 had the highest proportion; thus, it was the most important PP.

During the smoking process of SAV, the content of TWSP gradually increased and reached the maximum value of 95.45 ± 5.48 mg/100 g Pei on the 5th day (Table 2). The content of TWSP increased by 78.08% compared with that prior to smoking.

### 3.2. Changes in Physicochemical Indexes during the Smoking Process

**Figure 2:**
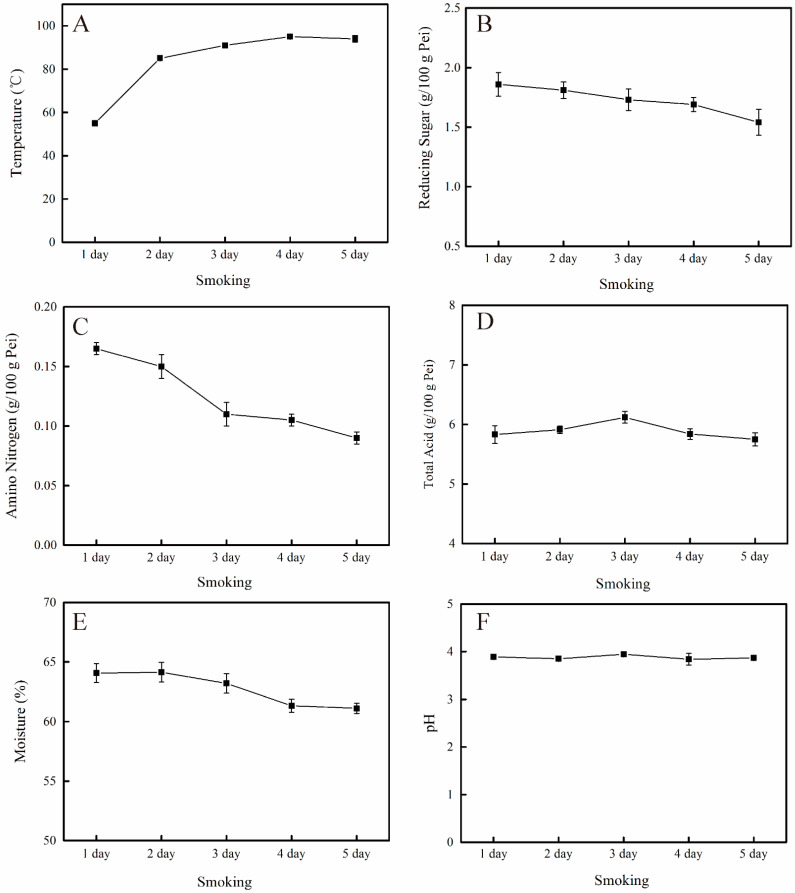
Changes in physicochemical indexes during the smoking process of Shanxi aged vinegar. (**A**–**F**) indicates the changes of temperature, reducing sugar, amino nitrogen, total acid, moisture and pH in the smoking process in turn.

The changes of physicochemical indexes during the smoking process were analyzed. Temperature initially increased, slightly decreased, and then reached the maximum of 95 °C on the 4th day (Figure 2A). The content of reducing sugars and amino nitrogen decreased by 17.20% and 45.45%, respectively (Figure 2B,C). The content of total acid initially increased, decreased, and then reached the maximum value of 6.12 ± 0.10 g/100 g Pei on the 3rd day (Figure 2D). The moisture content in smoking Pei ranged from 61% to 65% and decreased by 4.60% (Figure 2E). The pH fluctuated within the range of 3.8–4.0, but no obvious trend was observed (Figure 2F).

### 3.3. Analysis of Correlation among TWSP, PPs, and Environmental Factors during the Smoking Process

**Figure 3:**
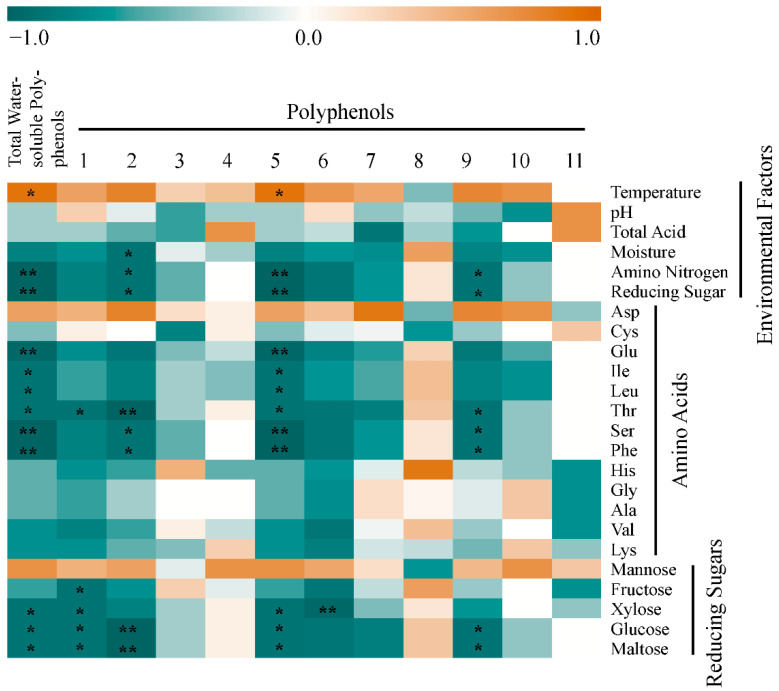
Spearman correlation analysis heat map between polyphenols and environmental factors during the smoking process of Shanxi aged vinegar (red and green indicate positive correlation and negative correlation, respectively, and the shade of color indicates the size of the correlation coefficient; * *p* < 0.05; ** *p* < 0.01).

Temperature, moisture, total acid, pH, total reducing sugars, and amino nitrogen are the main parameters for monitoring the smoking process. With physicochemical indexes as the environmental factors, Spearman correlation analysis was performed to analyze the correlation among TWSP, PPs, and environmental factors (Figure 3). The correlation between TWSP and environmental factors was analyzed. The environmental factors that are significantly correlated with TWSP were temperature, amino nitrogen, and reducing sugars (*p* < 0.05). By contrast, pH, total acid, and moisture content were not significantly correlated with TWSP (*p* > 0.05). Therefore, temperature, amino nitrogen, and reducing sugars may be the main environmental factors affecting the formation of TWSP. Temperature was positively correlated with TWSP, whereas amino nitrogen and reducing sugars were negatively correlated with TWSP. The correlation between these environmental factors and different types of PPs was analyzed. Results showed that PPs 2, 5, and 9 were significantly correlated with temperature, amino nitrogen, and reducing sugars. Temperature was significantly correlated with PP 5, whereas amino nitrogen and reducing sugars were significantly correlated with PPs 2, 5, and 9. Therefore, temperature, amino nitrogen, and reducing sugars may affect TWSP by affecting the production of PPs 2, 5, and 9. Moisture content was also significantly correlated with PP 2. Hence, it may also be one of the environmental factors affecting TWSP.

### 3.4. Analysis of Correlation among TWSP, PPs, and Amino Acids during the Smoking Process

Thirteen amino acids were detected during the smoking process of SAV: Asp, Thr, Ser, Glu, Gly, Ala, Cys, Val, Ile, Leu, Phe, His, and Lys (Supplementary Material Table S2). From the 1st day to the 5th day, the total amino acid content (calculated as amino acid nitrogen) was 0.113 ± 0.003, 0.092 ± 0.003, 0.082 ± 0.004, 0.081 ± 0.003, and 0.075 ± 0.004 g/100g Pei, respectively; the proportion of total amino acid content in amino nitrogen was 68.75%, 61.20%, 74.82%, 76.89%, and 82.99%, respectively (Figure 1, Supplementary Material Table S2). Therefore, amino acids were the main source of amino nitrogen. The total amino acid content during the smoking process decreased by 33.63% (Supplementary Material Table S2). Except for Asp and His, the content of most amino acids decreased after the smoking process. The amino acids that substantially decreased were Glu (100%), Thr (69.23%), Lys (65.63%), and Ser (65.38%) (Supplementary Material Table S2).

The correlation between amino acids, the supplier of amino nitrogen, and TWSP was analyzed. Six kinds of amino acids were significantly related to TWSP: Glu, Ile, Leu, Thr, Ser, and Phe (*p* < 0.05, Figure 3). Therefore, these amino acids may be the main amino acids that affect TWSP formation. Correlation analysis revealed that six kinds of amino acids (Glu, Ile, Leu, Thr, Ser, and Phe) and four kinds of PPs (1, 2, 5, and 9) were significantly correlated (*p* < 0.05, Figure 3). The types of amino acids were the same as those that affect TWSP. Therefore, these six amino acids may affect TWSP content by affecting the production of the four aforementioned PPs.

### 3.5. Analysis of Correlation among TWSP, PPs, and Reducing Sugars during the Smoking Process

Reducing sugars are another environmental factor that affects TWSP. Five kinds of reducing sugars were detected during the smoking process: xylose, fructose, mannose, glucose, and maltose (Supplementary Material Table S2). The content of xylose gradually decreased, whereas that of mannose, glucose, and maltose initially increased and then decreased. The change in the content of fructose did not show an obvious trend (Supplementary Material Table S2).

The correlation between reducing sugars and TWSP was analyzed. Three kinds of reducing sugars were significantly correlated with TWSP: xylose, glucose, and maltose (*p* < 0.05, Figure 3). Fructose and mannose were not significantly correlated with TWSP (p > 0.05, Figure 3). Therefore, xylose, glucose, and maltose may be the main reducing sugars affecting TWSP. Correlation analysis between reducing sugars and PPs revealed that four kinds of reducing sugars (xylose, glucose, maltose, and fructose) and five kinds of PPs (1, 2, 5, 6, and 9) were significantly correlated (Figure 3). Therefore, xylose, glucose, maltose, and fructose may affect the content of TWSP by affecting the production of the five aforementioned PPs. Xylose, glucose, and maltose were significantly related to TWSP and the five aforementioned PPs. Thus, they may be the main reducing sugars that affect TWSP (Figure 3). Fructose was also significantly correlated with PP 1. Hence, this reducing sugar may also be one of the factors affecting the content of total PPs (Figure 3).

### 3.6. Influence of Environmental Factors on TWSP 

The analyses in the previous sections suggested that temperature, amino nitrogen, and total reducing sugars may be the main environmental factors affecting TWSP. Six amino acids (Glu, Ile, Leu, Thr, Ser, and Phe) and three reducing sugars (xylose, glucose, and maltose) may be the main compounds that affect the content of TWSP. The influence of environmental factors on TWSP was verified by controlling the temperature and adding external sources. The externally added substances that may have a large effect on the content of TWSP were amino acids (Glu, Ile, Leu, Thr, Ser, and Phe) and reducing sugars (xylose, glucose, and maltose).

**Figure 4:**
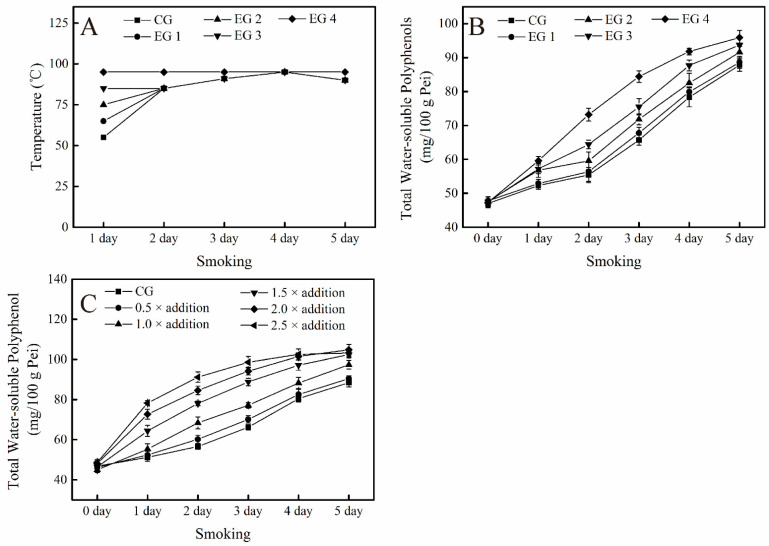
Single-factor experiment of environmental factors affecting the content of polyphenols: (**A**) temperature control process in temperature single factor experiment, (**B**) influence of temperature on the content of total water-soluble polyphenols, and (**C**) influence of external sources on the content of total water-soluble polyphenols.

For the experiment of the effect of temperature on TWSP, the temperature setting is shown in Figure 4A. The content of TWSP gradually increased during the smoking process. As temperature increased, the accumulation rate of TWSP increased (Figure 4B). At the end of the smoking process, the content of TWSP increased with the increase in temperature. By the end of the smoking process, the content of TWSP reached the maximum value of 95.9 ± 2.1 mg/100g Pei, which was an increase of 9.23% compared with that in the control group (Figure 4B). Exogenous addition of amino acids and reducing sugars also promoted the accumulation of TWSP (Figure 4C). The contents of amino acids and reducing sugars during the smoking process are given in Supplementary Material Table S2. As the amount of these substances added was increased, the production rate of TWSP also gradually increased. When the amount added is 0.5–1.5× of the initial concentration of the control group, the content of TWSP gradually increased. When the amount added was over 1.5 ×, the content of TWSP no longer increases (*p* > 0.05). Therefore, the content of TWSP reached the maximum value after the smoking process when the addition amount was 1.5 ×. In summary, increasing the temperature and the amount of amino acids and reducing sugars added increased the content of TWSP during the smoking process. Moreover, the content of TWSP reached the maximum value when the temperature was set to 95 °C and the addition ratio was 1.5×.

### 3.7. In situ Regulation of TWSP during the Smoking Process

**Figure 5:**
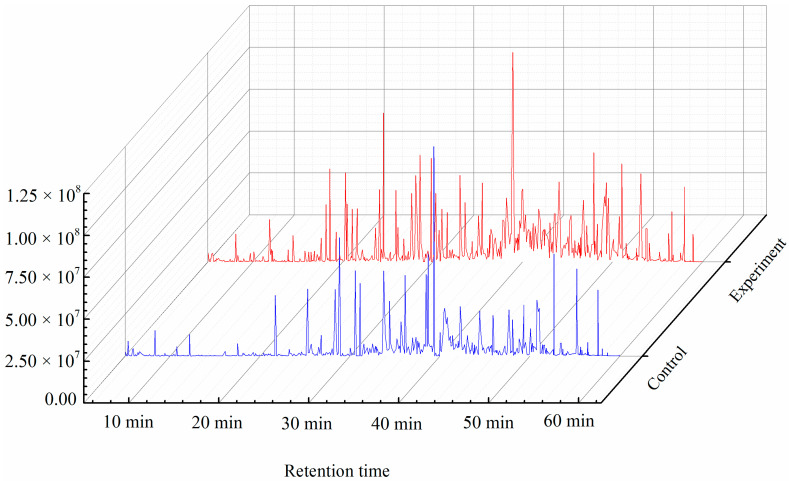
Comparison of gas chromatography–mass spectroscopy total ion diagram of in situ regulated and nonregulated smoking samples.

On the basis of the influence of environmental factors on the accumulation of TWSP, the content of TWSP during the smoking process was regulated in situ. The temperature was set to 95 °C, and the amount of amino acids (Glu, Ile, Leu, Thr, Ser, and Phe) and reducing sugars (xylose, glucose, and maltose) added was 1.5× of the initial concentration of the control group. The diagram of total ion current before and after regulation is shown in Figure 5. A total of 677 ± 11 substances were detected in the experimental group, which was higher than that in the control group (618 ± 25).

The types and contents of PPs during the smoking process remarkably changed after the regulation. Thirteen PPs were detected in the experimental group (Table 3). Aside from the eleven PPs in the control group (Table 1), (12) homoprotocatechuic acid and (13) catechin were also detected. These PPs were detected only on the first day of the smoking process. The distribution of PPs at the end of the smoking process after the regulation was analyzed. Similar to the control group, the number of PPs detected at the end of the smoking process in the experimental group was also nine. However, PP 8 disappeared in the experimental group and PP 10 was detected instead. As the main PPs in the control group, the content of PPs 1, 2, 3, 6, and 9 in the experimental group accounted for 6.77%, 14.04%, 37.26%, 23.12%, and 7.34%, respectively, for a total of 88.53% of the total amount of PPs. Therefore, PPs 1, 2, 3, 6, and 9 were also the main compounds in the experimental group. Similarly, PP 3 had the highest proportion, thus it was also the most important PP in the experimental group. Compared with that in the control group, the content of seven PPs, namely, PPs 1, 2, 3, 4, 5, 6, and 10, in the experimental group increased. Among them, the PPs with larger increments were PPs 3, 4, and 10, and their increments accounted for 81.90% of the TWSP increments. Therefore, the regulation strategy mainly promotes the production of PPs 2, 3, 4, and 10 to increase the accumulation of TWSP.

**Figure 6:**
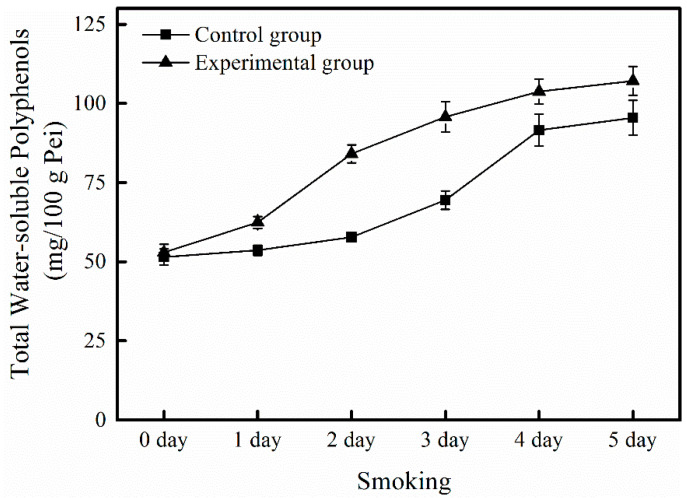
Effect of in situ regulation on changes in total water-soluble polyphenols content during the smoking process of Shanxi aged vinegar.

The absolute content of TWSP in the experimental group during the smoking process was analyzed. The slope of the curve in the experimental group was greater than that in the control group (Figure 6), indicating that the regulation accelerated the accumulation rate of TWSP during the smoking process. The content of TWSP gradually increased during the smoking process. On the 5th day, the content of TWSP in the experimental group reached the maximum value of 107.13 ± 4.55 mg/100 g Pei, an increase of 12.24% compared with that in the control group (the initial content of TWSP was 95.45±5.48 mg/100 g Pei) (*p* < 0.05, Figure 6). Therefore, this regulation strategy can substantially increase the production of TWSP.

## 4. Discussion

Smoking is a complex reaction system dominated by the Maillard reaction. PPs mainly accumulate during the smoking process and are affected by various environmental factors. In this study, the environmental factors affecting the accumulation of water-soluble PPs were analyzed and the regulation strategy of TWSP was proposed.

Many studies demonstrated that various reactions, such as degradation, oxidation loss, and melanoid formation in the Maillard reactions, reduce the content of PPs [13,24,25]. However, some conditions in the Maillard reaction may also promote the increase in the types and content of PPs, such as promoting the release, synthesis, and derivatization of PPs. The increase in temperature promoted the release of intracellular or bound PPs. The main PPs during the smoking process were phenolic acids (Table 1 and Table 3), which often exist in cells in soluble or insoluble form [26]. The increase in temperature destroyed the cell wall, thereby promoting the release of endogenous PPs. Phenolic acids are often coupled with cell wall polymers to form covalent complexes, such as PP 3, which is abundant in cereal cell walls [26]. The increase in temperature promotes the hydrolysis and release of bound phenolic acids under acidic conditions. The melanoids produced by the Maillard reaction are also rich in PPs [3,27]. The increase in temperature facilitates the breaking of the covalent [27] and noncovalent bonds (such as hydrogen bonds) [8] between polyphenols and melanoidin, as well as promotes the release of PPs. Therefore, the increase in the amount of PPs released may be one of the important reasons accounting for the increase in the accumulation of phenolic acids, such as PPs 3, 4, and 5 (Table 1 and Table 3). In addition to promoting the release of PPs, the increase in temperature also promotes the occurrence of some chemical reactions related to PPs. First of all, the increase in temperature may promote the accumulation of polyphenol precursors. High temperature denatures proteins and produces more free amino acids, and some of these amino acids, such as tyrosine [28], act as precursors to promote the synthesis of PPs. High temperature also increases the synthesis of the intermediates of the Maillard reaction, some of which are involved in the synthesis of PPs. For example, glyoxylic acid, as a provider of acetate groups, reacts with phenol to generate 4-hydroxyphenylacetic acid [29], which is beneficial to the production of PP 12. Secondly, the increase in temperature may promote the occurrence of some synthesis reactions. For example, the isomerization reaction of catechins [25] and the reaction of malonic acid and PP 1 to PP 3 [30]. Thirdly, the hydroxyl and aldehyde groups in PPs may be oxidized to carboxyl groups at high temperatures, thereby promoting the occurrence of oxidation reactions [15,31]. For example, the aldehyde group in PP 1 is oxidized to produce PP 2 [32], and induce the conversion between PP 8 and PP 7. In addition, (PP 5) 4-hydroxybenzoic acid and (PP 4) 4-hydroxycinnamic acid are the precursors for the synthesis of other hydroxybenzoic acid and hydroxycinnamic acid phenolic acids, respectively. Methoxylation at high temperatures promotes the formation of PP 1 and 3, respectively. In fact, PP 11 can also be converted to PP 3 [33]. The conversion of various PPs (such as PP 1, 4, and 11) to PP 3 may be the reason why PP 3 is the main PP during the smoking process of SAV (Table 1 and Table 3). The accumulation of some precursors, intermediates or products in the conversion reaction may be the reason for the significant correlation between environmental factors and them (such as PP 2, 5, etc.; see Figure 3).

The addition of amino acids and reducing sugars increased the substrate of the Maillard reaction and promoted the synthesis of PPs during the smoking process. Amino acids, especially aromatic amino acids, are important precursors for the synthesis of PPs. SAV is rich in numerous kinds of amino acids. The 4-hydroxybenzaldehyde synthesized from tyrosine [12] is the precursor for the synthesis of hydroxybenzoic phenolic acids, which can derive many polyphenols, such as PP 5 and PP 1. Phenylalanine is a common intermediate in the synthesis of PPs [26] and can derive various PPs [34]. Therefore, the addition of phenylalanine promotes the synthesis of PPs. In addition, some of the added amino acids are non-neutral amino acids, which may cause changes in environmental factors such as pH and affect the release of PPs. The reaction of amino acids and reducing sugars can also cause changes in some environmental factors. Therefore, the increase in the accumulation of PPs by exogenous addition may be due either to the direct addition of amino acids and reducing sugars or to other indirect factors derived from such addition. Sugars are an important source of polyphenol side chain groups. Glycolaldehyde, glyoxal, and glyoxylic acid are formed in large quantities in the Maillard reaction as the fragmentation products of sugars, providing aldehyde groups for PPs [35,36,37]. For example, glyoxylic acid reacts with guaiacol or catechol to form PP 1, and PP 7 is the intermediate product of the reaction [38,39]. Therefore, the increase in glyoxylic acid content is conducive to the production of PPs 1 and 7, and fluctuations in the content of PP 7 during the smoking process may be related to the conversion of PP 7 to PP 1 (Table 1).

However, changes in smoking conditions undeniably have a negative effect on PP accumulation. For example, catechins are easily degraded at high temperatures [25], which may be the reason why PPs 12 and 13 disappeared during the middle and late stages of smoking process (Table 3). High temperatures may cause more PPs to participate in the formation of melanoidin [24], and reduce the content of free PPs in vinegar. The effect of temperature on the bonding of PPs with the matrix is also related to the endothermic and exothermic heat of broken bonds. The increase in temperature is conducive to the hydrophobic interaction between the PPs and the matrix [8], which is not conducive to the release of PPs. In summary, the effects of promoting and restraining the accumulation of PPs during the smoking process occur simultaneously, and the promotion effect was dominant during the smoking process, which increased the content of TWSP.

In this study, Spearman’s correlation analysis and single factor experiment were used to screen environmental factors and verify, respectively. On the basis of the influence of environmental factors on TWSP, a regulation strategy for promoting the accumulation of TWSP was proposed from the perspective of strengthening the Maillard reaction. The distribution of PPs was first analyzed. A total of eleven PPs were detected during the smoking process of SAV. For the first time, temperature, amino acids, and reducing sugars were observed to promote the production of TWSP. By increasing the temperature and the content of amino acids and reducing sugars, the accumulation of TWSP increased by 12.24%. Although it has been discovered that environmental factors promote the accumulation of TWSP, their mechanism during the smoking process still needs further study. A simple model system eliminates the obstacles in analyzing complex reaction systems and is an effective means to reveal the formation mechanism of substances in complex systems [12,37]. In a follow-up study, we will comprehensively investigate the formation mechanism of PPs during the smoking process by simulating the conditions of smoking and constructing a reaction model.

## Figures and Tables

**Table 1:** Distribution of polyphenols during the smoking process of Shanxi aged vinegar.

Polyphenols	CAS Number	Compound Identification	Smoking				
			1 Day	2 Days	3 Days	4 Days	5 Days
1	121-33-5	4-Hydroxy-3-methoxy-benzaldehyde (Vanillin)	1.95 ± 0.19	1.04 ± 0.11	3.23 ± 0.13	2.70 ± 0.31	5.67 ± 0.14
2	121-34-6	4-Hydroxy-3-methoxy-benzoic acid (Vanillic acid)	3.65 ± 0.17	3.50 ± 0.22	5.44 ± 0.08	6.98 ± 0.19	10.43 ± 0.17
3	537-98-4	(2E)-3-(4-Hydroxy-3-methoxyphenyl)-2-propenoic acid ((E)-ferulicacid)	7.30 ± 0.36	13.07 ± 0.11	6.26 ± 0.23	11.37 ± 0.12	29.57 ± 0.16
4	501-98-4	(E)-3-(4-hydroxyphenyl)-2-propenoicacid ((E)-4-hydroxycinnamic acid)	4.55 ± 0.51	9.19 ± 0.04	10.44 ± 0.073	15.00 ± 0.30	27.01 ± 0.11
5	99-96-7	4-Hydroxybenzoic acid	0	0.40 ± 0.13	0.78 ± 0.31	0.91 ± 0.06	1.26 ± 0.01
6	93376-04-6	(3R,4R)-Dihydro-3,4-bis[(3-hydroxy-4-methoxyphenyl) methyl]-2(3H)-furanone	0	0	13.14 ± 0.14	11.74 ± 0.12	19.99 ± 0.14
7	55-10-7	4-Hydroxy-3-methoxymandelic acid (Vanillinmandelic acid)	0.57 ± 0.18	0	0	1.18 ± 0.18	2.07 ± 0.07
8	40979-91-7	1-(3-Hydroxy-4-methoxyphenyl)-1,2-ethanediol	0.37 ± 0.19	10.85 ± 0.17	0	0	3.73 ± 0.21
9	99-10-5	3,5-Dihydroxybenzoic acid	0	0	0	5.58 ± 0.25	12.69 ± 0.31
10	96251-92-2	(E)-3-(3-hydroxyphenyl) acrylic acid ethyl ester	0	0	0	7.03 ± 0.15	0
11	1135-23-5	3-(4-Hydroxy-3-methoxyphenyl) propionic acid (Dihydroferulic acid)	0	0	1.03 ± 0.15	0	0

CAS, Chemical Abstracts Service.

**Table 2:** Changes in total water-soluble polyphenols during the smoking process of Shanxi aged vinegar.

Name	Smoking				
	1 Day	2 Days	3 Days	4 Days	5 Days
Total water-soluble polyphenols (mg/100 g Pei)	53.60 ± 1.57	57.78 ± 1.01	69.47 ± 2.93	91.59 ± 5.00	95.45 ± 5.48

**Table 3:** Effect of in situ regulation on the distribution of polyphenols during the smoking process of Shanxi aged vinegar.

Polyphenols	CAS Number	Compound Identification	Smoking				
			1 Day	2 Days	3 Days	4 Days	5 Days
1	121-33-5	4-Hydroxy-3-methoxy-benzaldehyde (Vanillin)	2.34 ± 0.21	2.58 ± 0.16	4.34 ± 0.18	4.62 ± 0.26	6.82 ± 0.25
2	121-34-6	4-Hydroxy-3-methoxy-benzoic acid (Vanillic acid)	3.15 ± 0.26	4.24 ± 0.17	6.47 ± 0.28	9.78 ± 0.39	14.14 ± 0.47
3	537-98-4	(2E)-3-(4-Hydroxy-3-methoxyphenyl)-2-propenoic acid ((E)-ferulicacid)	7.77 ± 0.39	16.27 ± 0.22	29.23 ± 0.29	32.31 ± 0.42	37.52 ± 0.53
4	501-98-4	(E)-3-(4-hydroxyphenyl)-2-propenoicacid ((E)-4-hydroxycinnamic acid)	3.23 ± 0.18	5.19 ± 0.24	6.62 ± 0.14	7.43 ± 0.36	7.33 ± 0.32
5	99-96-7	4-Hydroxybenzoic acid	0.66 ± 0.21	0.74 ± 0.16	0.83 ± 0.25	1.21 ± 0.32	1.76 ± 0.22
6	93376-04-6	(3R,4R)-Dihydro-3,4-bis[(3-hydroxy-4-methoxyphenyl) methyl]-2(3H)-furanone	0	7.78 ± 0.31	11.27 ± 0.19	22.14 ± 0.33	23.28 ± 0.43
7	55-10-7	4-Hydroxy-3-methoxymandelic acid (Vanillinmandelic acid)	0	0	0	0	1.39 ± 0.12
8	40979-91-7	1-(3-Hydroxy-4-methoxyphenyl)-1,2-ethanediol	1.26 ± 0.14	0.18 ± 0.14	0	0	0
9	99-10-5	3,5-Dihydroxybenzoic acid	3.11 ± 0.17	3.88 ± 0.26	3.61 ± 0.13	4.44 ± 0.32	7.39 ± 0.22
10	96251-92-2	(E)-3-(3-hydroxyphenyl) acrylic acid ethyl ester	0	0	0	1.15 ± 0.17	1.07 ± 0.14
11	1135-23-5	3-(4-Hydroxy-3-methoxyphenyl) propionic acid (Dihydroferulic acid)	3.24 ± 0.16	6.26 ± 0.33	5.44 ± 0.36	0	0
12	102-32-9	3,4-Dihydroxybenzeneacetic acid (Homoprotocatechuic acid)	9.02 ± 0.20	0	0	0	0
13	154-23-4	(2R,3S)-2-(3,4-Dihydroxyphenyl)-3,4-dihydro-2H-1-benzopyran-3,5,7-triol (Catechin)	0.19 ± 0.04	0	0	0	0

CAS, Chemical Abstracts Service.

## Data Availability

No data was reported in this study.

## Supplementary Materials

The following are available online at https://www.mdpi.com/article/10.3390/foods10071518/s1. Table S1: Structural formula of polyphenols during the smoking process of Shanxi aged vinegar. Table S2: Variations of types and contents of amino acids and reducing sugars during the smoking process of Shanxi aged vinegar.
