# Supplementary material for: Elucidation and Regulation of Polyphenols in the Smoking Process of Shanxi Aged Vinegar
Source: Foods. 2021 Jul 1;10(7):1518. doi: 10.3390/foods10071518 (PMC8305182; doi:10.3390/foods10071518)
Supplement: Supplementary file 1 [file foods-10-01518-s001.zip › foods-1242225-supplementary.pdf]

Table S1. Structural formula of polyphenols during the smoking process of Shanxi aged vinegar.

Polyphenols	CAS Number	Compound Identification	Structure
1	121-33-5	4-Hydroxy-3-methoxy-benzaldehyde (Vanillin)	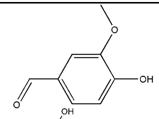
2	121-34-6	4-Hydroxy-3-methoxy-benzoic acid (Vanillic acid)	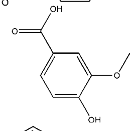
3	537-98-4	(2E)-3-(4-Hydroxy-3-methoxyphenyl)-2-propenoic acid ((E)-Ferulicacid)	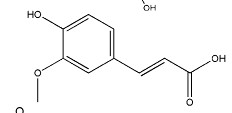
4	501-98-4	(e)-3-(4-hydroxyphenyl)-2-propenoicacid ((E)-4-Hydroxycinnamic acid)	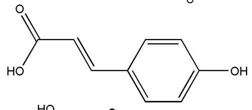
5	99-96-7	4-Hydroxybenzoic acid	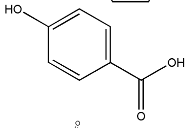
6	93376-04-6	(3R,4R)-Dihydro-3,4-bis[(3-hydroxy-4-methoxyphenyl) methyl]-2(3H)-furanone	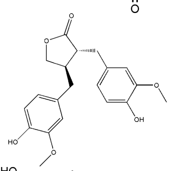
7	55-10-7	4-Hydroxy-3-methoxymandelic acid	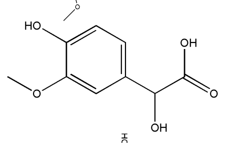
8	40979-91-7	1-(3-Hydroxy-4-methoxyphenyl)-1,2-ethanediol	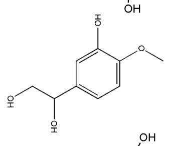
9	99-10-5	3,5-Dihydroxybenzoic acid	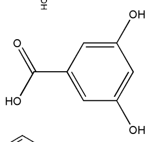
10	3943-96-2	(E)-3-(3-hydroxyphenyl) acrylic acid ethyl ester	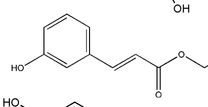
11	1135-23-5	3-(4-Hydroxy-3-methoxyphenyl) propionic acid (Dihydroferulic acid)	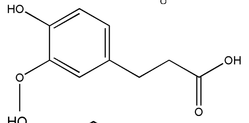
12	102-32-9	3,4-Dihydroxybenzeneacetic acid (Homoprotocatechuic acid)	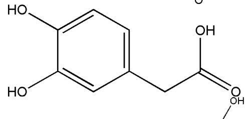
13	154-23-4	(2R,3S)-2-(3,4-Dihydroxyphenyl)-3,4-dihydro-2H-1-benzopyran-3,5,7-triol (Catechin)	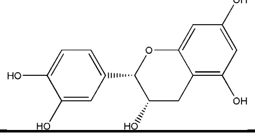

Table S2. Variations of types and contents of amino acids and reducing sugars during the smoking process of Shanxi aged vinegar.

Compounds	Smoking (mg/g Pei)				
	1 day	2 day	3 day	4 day	5 day
Asp	0.44 ± 0.06	0.41 ± 0.01	0.42 ± 0.02	0.54 ± 0.01	0.49 ± 0.02
Thr	0.39 ± 0.01	0.41 ± 0.05	0.36 ± 0.03	0.13 ± 0.03	0.12 ± 0.05
Ser	0.52 ± 0.01	0.32 ± 0.02	0.31 ± 0.03	0.28 ± 0.04	0.18 ± 0.05
Glu	0.97 ± 0.04	0.80 ± 0.03	0.58 ± 0.02	0	0
Gly	0.37 ± 0.01	0.31 ± 0.03	0.27 ± 0.02	0.36 ± 0.05	0.31 ± 0.03
Ala	1.73 ± 0.03	1.49 ± 0.04	1.19 ± 0.05	1.52 ± 0.02	1.42 ± 0.02
Cys	1.36 ± 0.12	0.94 ± 0.01	1.20 ± 0.04	1.19 ± 0.02	1.11 ± 0.02
Val	0.84 ± 0.01	0.68 ± 0.03	0.59 ± 0.04	0.64 ± 0.02	0.63 ± 0.03
Ile	0.51 ± 0.01	0.38 ± 0.03	0.35 ± 0.04	0.31 ± 0.01	0.32 ± 0.04
Leu	1.00 ± 0.03	0.78 ± 0.03	0.66 ± 0.05	0.46 ± 0.04	0.53 ± 0.05
Phe	0.49 ± 0.02	0.38 ± 0.01	0.28 ± 0.05	0.26 ± 0.03	0.26 ± 0.05
His	0.55 ± 0.04	0.56 ± 0.01	0.49 ± 0.04	0.49 ± 0.05	0.55 ± 0.03
Lys	0.32 ± 0.02	0.21 ± 0.05	0.19 ± 0.04	0.31 ± 0.04	0.11 ± 0.01
Xylose	15.43 ± 0.55	14.05 ± 0.46	13.01 ± 0.02	12.76 ± 0.23	12.95 ± 0.24
Fructose	7.79 ± 0.03	7.87 ± 0.02	7.68 ± 0.01	7.70 ± 0.04	7.69 ± 0.01
Mannose	10.18 ± 0.04	11.35 ± 0.42	11.77 ± 0.18	13.09 ± 0.18	11.49 ± 0.55
Glucose	1.08 ± 0.04	1.80 ± 0.02	0.70 ± 0.01	0.66 ± 0.01	0.63 ± 0.01
Maltose	26.63 ± 0.19	30.94 ± 0.20	23.50 ± 0.09	23.38 ± 0.01	23.37 ± 0.01
